# Molecular imaging analysis of microvesicular and macrovesicular lipid droplets in non-alcoholic fatty liver disease by Raman microscopy

**DOI:** 10.1038/s41598-020-75604-6

**Published:** 2020-10-29

**Authors:** Takeo Minamikawa, Mayuko Ichimura-Shimizu, Hiroki Takanari, Yuki Morimoto, Ryosuke Shiomi, Hiroki Tanioka, Eiji Hase, Takeshi Yasui, Koichi Tsuneyama

**Affiliations:** 1 Department of Post-LED Photonics Research, Institute of Post-LED Photonics, Tokushima University, 2-1 Minami-Josanjima, Tokushima, 770-8506 Japan; 2 Graduate School of Technology, Industrial and Social Sciences, Tokushima University, 2-1 Minami-Josanjima, Tokushima, Tokushima 770-8506 Japan; 3 PRESTO, Japan Science and Technology Agency (JST), 2-1 Minami-Josanjima, Tokushima, Tokushima 770-8506 Japan; 4 Research Cluster On “Multi-Scale Vibrational Microscopy for Comprehensive Diagnosis and Treatment of Cancer”, Tokushima University, 2-1 Minami-Josanjima, Tokushima, Tokushima 770-8506 Japan; 5 Department of Pathology and Laboratory Medicine, Graduate School of Medical Sciences, Tokushima University, 3-18-15 Kuramoto, Tokushima, Tokushima 770-8503 Japan; 6 Department of Interdisciplinary Researches for Medicine and Photonics, Institute of Post-LED Photonics, Tokushima University, 3-18-15 Kuramoto, Tokushima, Tokushima 770-8503 Japan

**Keywords:** Fats, Non-alcoholic fatty liver disease, Non-alcoholic steatohepatitis, Molecular imaging, Imaging and sensing, Raman spectroscopy

## Abstract

Predominant evidence of non-alcoholic fatty liver disease (NAFLD) is the accumulation of excess lipids in the liver. A small group with NAFLD may have a more serious condition named non-alcoholic steatohepatitis (NASH). However, there is a lack of investigation of the accumulated lipids with spatial and molecular information. Raman microscopy has the potential to characterise molecular species and structures of lipids based on molecular vibration and can achieve high spatial resolution at the organelle level. In this study, we aim to demonstrate the feasibility of Raman microscopy for the investigation of NAFLD based on the molecular features of accumulated lipids. By applying the Raman microscopy to the liver of the NASH model mice, we succeeded in visualising the distribution of lipid droplets (LDs) in hepatocytes. The detailed analysis of Raman spectra revealed the difference of molecular structural features of the LDs, such as the degree of saturation of lipids in the LDs. We also found that the inhomogeneous distribution of cholesterol in the LDs depending on the histology of lipid accumulation. We visualised and characterised the lipids of NASH model mice by Raman microscopy at organelle level. Our findings demonstrated that the Raman imaging analysis was feasible to characterise the NAFLD in terms of the molecular species and structures of lipids.

## Introduction

Non-alcoholic fatty liver disease (NAFLD) is the most common liver disease associated with the accumulation of excess lipids in the liver^1^. The prevalence of NAFLD was increased worldwide and estimated to be 20–30% of the global population^2^. NAFLD increases the risk of cirrhosis and liver cancer, and it is a disease with poor outcomes and limited therapeutic options in advanced stages. Furthermore, the clinical burden of NAFLD is not only in the liver dysfunctions but affects extra-hepatic organs and regulatory pathways^3^. Therefore, the development of diagnostic methods at an early stage and the understanding of the pathogenesis of NAFLD has become a serious clinical concern worldwide.

NAFLD can be classified into two types, i.e., non-alcoholic steatohepatitis (NASH) that leads to liver-related complications such as cirrhosis and hepatocellular carcinoma, and non-alcoholic fatty liver (NAFL) that basically has benign prognosis. Although several hypotheses were proposed to describe the pathogenesis of the NAFLD, the pathogenesis of the progression to NASH or NAFL is still under investigation. Moreover, NASH and NAFL at the early stage exhibit similar evidence of the accumulation of excess lipids without apparent inflammation and fibrosis, making diagnosis difficult. In the current studies, NAFLD was predominantly investigated in terms of protein or gene activities in the liver. Although the predominant evidence of NAFLD is the accumulation of excess lipids, the pathological role of the lipids on NAFLD has not been clearly understood due to a lack of lipid-based investigation of NAFLD.

To investigate NAFLD in terms of accumulated lipids, some visualisation methods of lipid droplets (LDs) were proposed, i.e., dye-based visualisation methods^4,5^, imaging mass spectrometry^6,7^ and Raman microscopy^8,9^. Among these methods, Raman microscopy is a good candidate for the investigation of the accumulated lipids based on molecular species and structures of lipids. Raman microscopy measures Raman spectra that reflect molecular vibrations of intrinsic molecules. Since the molecular vibrations are sensitive to the species of atoms and chemical bonds of molecules, Raman spectroscopy provides information about the molecular species and structures via molecular vibrations. As visible and tightly focused excitation laser can be used for observation, high spatial resolution down to 500 nm or smaller can be realized. Furthermore, it has the potential to utilize in vivo evaluation owing to the visible light usage. Raman microscopy is, therefore, a powerful tool for the investigation of lipids in cells and tissues^10–15^. Although some researchers applied the Raman microscopy to NAFLD^16–18^, the efficacy of Raman microscopy for the investigation of NAFLD is still under investigation.

In the present study, we seek to provide a proof-of-principle demonstration of Raman microscopy in the investigation of NAFLD. Especially, we visualise and characterise the accumulated lipids in the liver of mice by Raman microscopy for the investigation of NAFLD in terms of molecular species and structures of lipids.

## Results

### Raman spectra of liver tissues of NASH model mice

**Figure 1:**
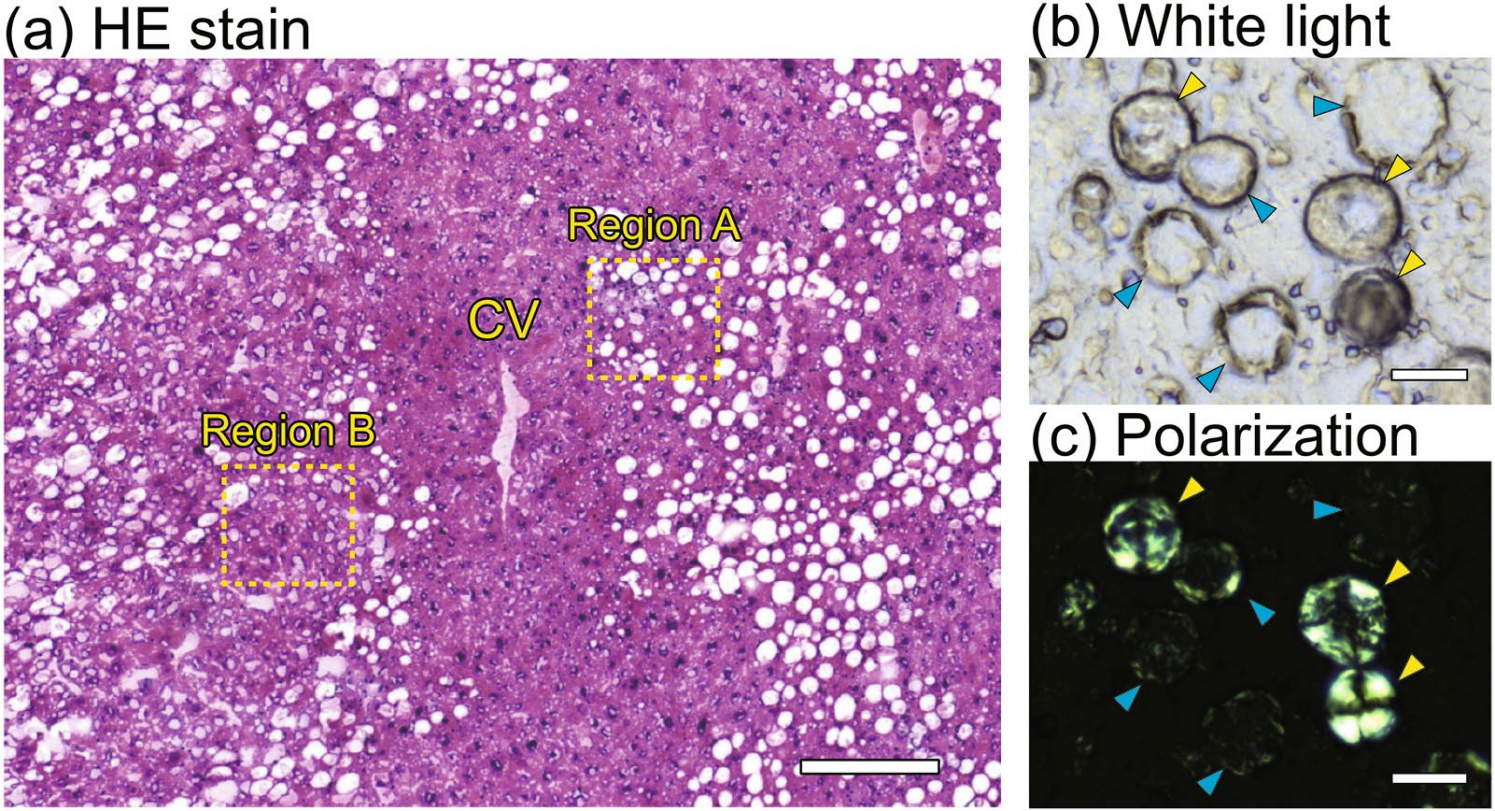
Typical histology of the liver tissue of a NASH model mouse. (**a**) HE-stained image of the NASH liver around a central vein (CV). (**b**) White light image of macrovesicular LDs of the NASH liver without any fixation nor staining. (**c**) Polarization image of the macrovesicular LDs of the NASH liver with cross-Nicol configuration. Blue arrowheads indicate LDs with an amorphous form of lipids. Yellow arrowheads indicate LDs with a crystalline form of lipids. Scale bars of (**a**), (**b**), and (**c**) indicate 200 µm, 10 µm, and 10 µm, respectively.

Typical Raman spectra of liver tissues of NASH model mice were obtained to reveal the spectral features for Raman spectral analysis. We focused on macrovesicular LDs accumulated near a central vein, as shown in region A in Fig. 1a. The previous studies had shown that the crystallinity of LDs is an important indicator of NAFLD prognosis^19^. By using the conventional polarization imaging with cross-Nicol configuration, the crystallinity of lipids in LDs can be identified as shown in Fig. 1b,c. We can observe a Maltese cross appearance owing to the polarization modulation by birefringence of a crystalline form of lipids, as indicated by the yellow arrowheads in Fig. 1c. If the Maltese cross is not apparent as indicated by the blue arrowhead in Fig. 1c, the LDs might predominantly contain an amorphous form of lipids. Thus, we investigate the spectral features of the Raman spectra of accumulated LDs with crystalline and amorphous forms of lipids in addition to hepatocytes with non-apparent LDs.

**Figure 2:**
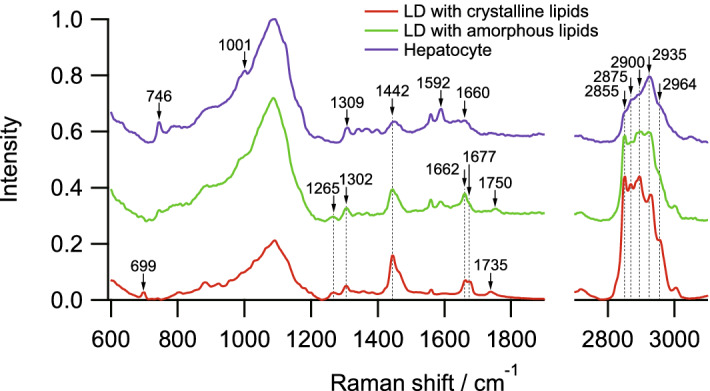
Typical Raman spectra of LDs with crystalline and amorphous forms of lipids and hepatocyte of the liver tissue of a NASH model mouse. The intensity was normalized by the highest intensities of the Raman spectra. LD, lipid droplet.

In the crystalline lipid-rich LDs, main Raman bands were found at 699, 1265, 1302, 1442, 1662, 1677, 1735, 2855, 2875, 2900, 2935, and 2964 cm^−1^ as shown in Fig. 2 and Table 1. The Raman bands at 1265, 1302, 1442, 2855, and 2900 cm^−1^ were assigned to CH_2_-related molecular bonds, indicating the presence of lipid contents in this region. The Raman band at 1662 cm^−1^ was assigned to C=C double bond stretching mode at the acyl chain, which is an indicator of the unsaturation of the acyl chain of lipids. The Raman bands at 699 and 1677 cm^−1^ respectively assigned to steroid ring vibration mode and C=C double bond stretching mode at carbon ring, indicating the presence of cholesterol content. As a result, the Raman spectra at the crystalline lipid-rich LDs reflected the molecular features of the LDs, including the cholesterols. The presence of cholesterol in the crystalline lipid-rich LDs was well agreed with the results of the fluorescence imaging with a cholesterol-specific dye in the previous studies^19^.

**Table 1:** Assignments of the Raman bands of LDs with crystalline and amorphous forms of lipids and hepatocyte of the liver tissue of a NASH model mouse.

Assignment	LDs (crystalline)	LDs (amorphous)	Hepatocyte	Main contribution
Steroid rings stretch	699			Cholesterol
Pyrrole breathing			746	Heme
Si–O vibrations	900–1200	900–1200	900–1200	Background of silica slide glass
Symmetric ring breath in phenylalanine			1001	Phenylalanine
=C–H bend	1265	1265		Lipids
CH_2_ twist	1302	1302		Lipids
All heme bonds			1309	Heme
CH_2_ bend	1442	1442	1448	Lipids
Redox and spin state of heme			1592	Heme
Amide I			1640–1680	Proteins
C=C stretch in acyl chain	1662	1662		Lipids
C=C stretch in carbon ring	1677			Cholesterol
C=O stretch	1735	1750		Lipids
CH_2_ symmetric stretch	2855	2855	2855	Lipids
CH_2_ asymmetric stretch	2875	2875	2875	Lipids
Fermi resonance CH_2_ stretch	2900	2900	2900	Lipids
CH_3_ symmetric stretch	2935	2935	2935	Proteins
CH_3_ asymmetric stretch	2964	2964	2964	Proteins

In the amorphous lipid-rich LDs, main Raman bands were found at 1265, 1302, 1442, 1662, 1750, 2855, 2875, 2900, 2935, and 2964 cm^−1^ as shown in Fig. 2 and Table 1. The main Raman bands were similar to that at the crystalline lipid-rich LDs, but the difference was found at 699 and 1677 cm^−1^, indicating relatively low cholesterol content at the LDs in terms of Raman spectral analysis. This result is also agreed with the result of the fluorescence imaging in the previous studies^19^. Importantly, Raman microscopy additionally has the potential to quantitatively evaluate the relative content of molecular species and structures without staining by analysing the relative intensity of each Raman band.

In contrast, in the hepatocytes with non-apparent LDs, main Raman bands were found at 746, 1001, 1309, 1448, 1592, 1660, 2855, 2875, 2900, 2935 and 2964 cm^−1^ as shown in Fig. 2 and Table 1. The Raman spectrum of the hepatocytes was different from that of these two types of LDs. Especially, heme-related Raman bands at 746, 1309, and 1592 cm^−1^ were observed in the hepatocytes, which might be derived by cytochromes present in mitochondria in hepatocytes or deposited hemoglobin. As a result, the Raman bands of LDs can be confirmed against that of hepatocytes, indicating the Raman spectral analysis of LDs can be performed in the liver tissues of the NASH model mice.

### Raman spectral imaging of macrovesicular LDs accumulated in hepatocytes

**Figure 3:**
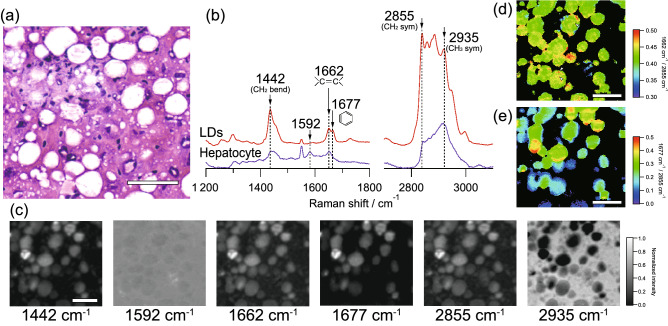
Raman spectral imaging of macrovesicular LDs accumulated in hepatocyte of liver tissues of NASH model mice. (**a**) HE-stained image of macrovesicular LDs indicated by region A in Fig. 1a. Raman imaging was performed on an unstained tissue section corresponding to the same position as the HE-stained image. (**b**) Typical Raman spectra of LDs and hepatocytes in this region. (**c**) Raman images of typical Raman bands. (**d**) Intensity ratio imaging of Raman bands of 1662 and 2855 cm^−1^, indicating the unsaturation degree of LDs. (**e**) Intensity ratio imaging of Raman bands of 1677 and 2855 cm^−1^, indicating the relative content of cholesterol. These intensity ratio images were obtained at the region with enough high intensity at the Raman band of 2855 cm^−1^, while the other region was masked. Scale bars of (**a**), (**c**), (**d**), and (**e**) indicate 50 µm.

We also investigated the molecular contents of lipids in terms of the size of LDs. The previous studies had shown that the size of LDs is closely related to the prognosis of NAFLD^20^. Firstly, we investigated the spatial distribution of molecular contents of lipids in macrovesicular LDs accumulated in hepatocyte of liver tissues of NASH model mice. We obtained two-dimensional Raman images of macrovesicular LDs in region A in Fig. 1a. In this region, macrovesicular LDs with about 10–40 µm in diameter were diffusely distributed, as shown in Fig. 3a. The typical Raman spectra of LDs and hepatocytes in this region were shown in Fig. 3b. As similar results of Fig. 2, the Raman spectrum of LDs was predominantly composed of lipid-related Raman bands, such as 1442, 1662, 1677, and 2855 cm^−1^, as shown in Fig. 3b. Furthermore, cholesterol-related Raman bands such as 1677 cm^−1^ were also observed. Raman images of each typical Raman bands were shown in Fig. 3c. The lipid-related Raman bands, such as 1442, 1662, 1677, and 2855 cm^−1^, clearly reflect the distribution of LDs, which are well agreed with the histology of the liver tissues of the NASH model mice confirmed by the HE-stain image. As the imaging analysis, each LD seems to be separated from each other. In contrast, the Raman bands at 1592 and 2935 cm^−1^ visualised the distribution of hepatocytes or heme proteins.

To analyse the more specific molecular species and structures of LDs, we performed intensity ratio imaging by using lipid-related Raman bands. The intensity ratio calculation was performed at each pixel to obtain relative contributions of lipid species and structures with the normalization of the amount of lipids. Firstly, we obtained an intensity ratio of 1662 cm^−1^ against 2855 cm^−1^, as shown in Fig. 3d, which indicates the unsaturation degrees of lipids. The macrovesicular LDs were exhibited inhomogeneous distribution of the intensity ratio of 1662 cm^−1^ against 2855 cm^−1^. Notably, some particulate structures of the intensity ratio were found in the internal structure of LDs, as typically indicated by the arrowheads in Fig. 3d. These results might indicate the inhomogeneous distribution of lipids according to unsaturation degrees of lipids, even in the macrovesicular LDs with morphologically uniform distribution.

We also obtained the intensity ratio of 1677 cm^−1^ against 2855 cm^−1^, as shown in Fig. 3e, which indicates the relative content of cholesterol. We found that the inhomogeneous distribution of the intensity ratio of 1677 cm^−1^ against 2855 cm^−1^ among LDs. In addition, some of the LDs exhibited a high-intensity ratio partially on the outer wall of the LDs. These results might indicate that the relative content of cholesterol was varied depending on the macrovesicular LDs, and the cholesterol distribution was unevenly distributed even in a single macrovesicular LD.

### Raman spectral imaging of microvesicular LDs accumulated in hepatocytes

**Figure 4:**
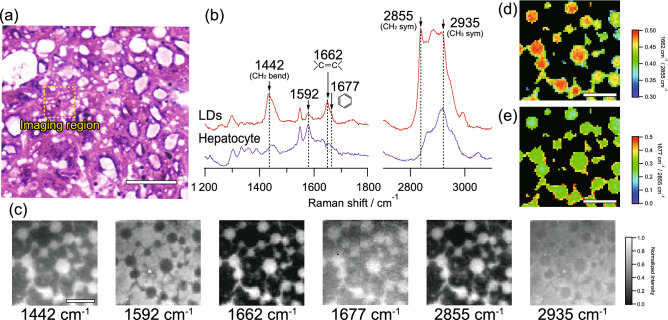
Raman spectral imaging of microvesicular LDs accumulated in hepatocyte of liver tissues of NASH model mice. (**a**) HE-stained image of microvesicular LDs indicated by region B in Fig. 1a. Raman imaging was performed on an unstained tissue section corresponding to the dashed squared region of the HE-stained image. (**b**) Typical Raman spectra of LDs and hepatocytes in this region. (**c**) Raman images of typical Raman bands. (**d**) Intensity ratio imaging of Raman bands of 1662 and 2855 cm^−1^, indicating the unsaturation degree of LDs. (**e**) Intensity ratio imaging of Raman bands of 1677 and 2855 cm^−1^, indicating the relative content of cholesterol. These intensity ratio images were obtained at the region with enough high intensity at the Raman band of 2855 cm^−1^, while the other region was masked. Scale bars of (**a**), (**c**), (**d**), and (**e**) indicate 50 µm, 10 µm, 10 µm, and 10 µm, respectively.

We also investigated the spatial distribution of molecular contents of lipids in microvesicular LDs accumulated in hepatocyte of liver tissues of NASH model mice. We obtained two-dimensional Raman images of macrovesicular LDs at the dashed squared region of Fig. 4a that are in region B in Fig. 1a. In this region, microvesicular LDs with about a few micrometers or less in diameter were diffusely distributed, as shown in Fig. 4a. The typical Raman spectra of LDs and hepatocytes in this region were shown in Fig. 4b. The Raman spectrum of LDs was exhibited similar to the macrovesicular LDs, such as 1442, 1662, 1677, and 2855 cm^−1^, as shown in Fig. 3b. However, the relative contribution of 1677 cm^−1^ was much smaller than that of macrovesicular LDs. Raman images of each typical Raman bands were shown in Fig. 4c. The lipid-related Raman bands, such as 1442, 1662, 1677, and 2855 cm^−1^, reflect the distribution of LDs, but interestingly, we found the connecting structure between LDs, which was not apparent in the HE-stained image. In contrast, the Raman bands at 1592 and 2935 cm^−1^ visualised the distribution of hepatocytes or heme proteins as similar to the result around the macrovesicular LDs.

Intensity ratio imaging was also performed by using lipid-related Raman bands. We obtained the intensity ratio of 1662 cm^−1^ against 2855 cm^−1^, as shown in Fig. 4d, which indicates the unsaturation degrees of lipids. The microvesicular LDs have exhibited an almost homogeneous distribution of the intensity ratio of 1662 cm^−1^ against 2855 cm^−1^. We also obtained the intensity ratio of 1677 cm^−1^ against 2855 cm^−1^, as shown in Fig. 4e, which indicates the relative content of cholesterol. The distribution of the intensity ratio of 1677 cm^−1^ against 2855 cm^−1^ was also homogeneous. These results might indicate the unsaturation degree of microvesicular LDs, and the relative content of cholesterol could distribute homogeneously in microvesicular LDs.

### Statistical comparison of molecular species and structural features of LDs between microvesicular and macrovesicular LDs

**Figure 5:**
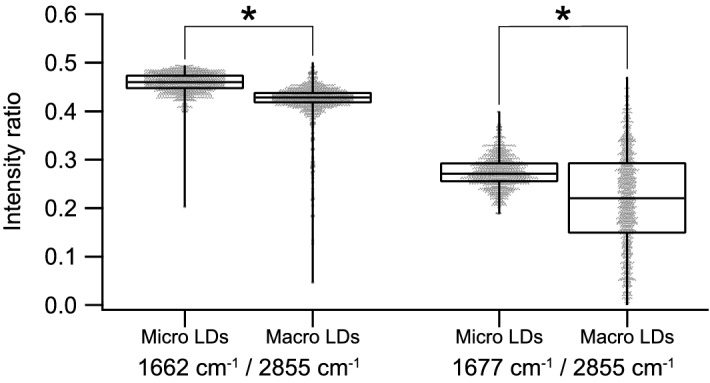
Box plot analysis of intensity ratio of Raman bands indicating the unsaturation degree of lipids (1662 cm^−1^/2855 cm^−1^) and the relative content of cholesterol (1677 cm^−1^/2855 cm^−1^) of microvesicular LDs (Micro LDs) and macrovesicular LDs (Macro LDs). A total of 1270 points for each of 21 microvesicular LDs and 37 macrovesicular LDs were evaluated. The individual data are shown by dots. *, significant difference (p < 0.01).

Finally, we investigated the difference of molecular species and structural features of LDs between microvesicular and macrovesicular LDs. We evaluated the intensity ratio of 1662 cm^−1^ against 2855 cm^−1^ and the that of 1677 cm^−1^ against 2855 cm^−1^ of the central region of the 37 LDs in macrovesicular LDs and 21 LDs in microvesicular LDs. The intensity ratio analyses of these Raman bands were shown in Fig. 5. The mean intensity ratio of 1662 cm^−1^ against 2855 cm^−1^ of the microvesicular LDs (0.438 ± 0.038) was significantly higher than that of the macrovesicular LDs (0.425 ± 0.034). This result shows the microvesicular LDs have a higher unsaturation degree of lipids than the macrovesicular LDs. This tendency was also observed by the other indicator of unsaturation degree^17^ (intensity ratio of 1265 against 1442 cm^−1^ and 3012 against 2855 cm^−1^), as shown in Supplementary Fig. 3. The mean intensity ratio of 1677 cm^−1^ against 2855 cm^−1^ of the microvesicular LDs (0.308 ± 0.061) was also significantly higher than that of the macrovesicular LDs (0.218 ± 0.104). This result shows the microvesicular LDs have a higher cholesterol content on average. However, the highest intensity ratio of 1677 cm^−1^ against 2855 cm^−1^ was found in the macrovesicular LDs, showing the partial aggregation of a higher content of cholesterol.

## Discussion

In the present study, we evaluated liver tissues of NASH model mice in terms of the molecular analysis of LDs within hepatocytes. We analysed spectral features of the Raman spectra of LDs with crystalline and amorphous forms of lipids and hepatocytes. We also successfully visualised the LDs according to the molecular species and structural features of lipids in the microvesicular and macrovesicular LDs. The Raman microscopy reveals the complicated distributions of lipids in LDs, which cannot be identified by conventional histological imaging techniques. Our results provided a proof-of-principle for the investigation of NAFLD using Raman microscopy in terms of the molecular species and structures of accumulated lipids in NAFLD.

NAFLD induces hepatocyte steatosis and liver dysfunction with glycolipid metabolic disorders based on insulin resistance. The pathogenesis is thought to be due to various factors such as oxidative stress, endoplasmic reticulum stress, autophagy, lipotoxicity owing to free fatty acids, and innate immune activation associated with bacterial intestinal abnormalities^21–23^; accordingly, various researches on NAFLD were conducted in terms of genome-wide association studies^24^, molecular biological studies^22,25,26^, and medical studies in both the aspect of treatment and diagnosis^27,28^. As regarding lipids, abnormal lipid metabolism and the induction of inflammation by overproduction of free fatty acids have been widely investigated. However, the current studies related to lipids have been predominantly performed by ensemble-averaged analysis of lipids without spatial information or by morphological analysis of accumulated LDs without molecular information. Since the accumulation of excess lipids is fundamental evidence of NAFLD, the analysis of lipids with both spatial and molecular information must provide an essential perspective in elucidating the pathogenesis of NAFLD. Our results demonstrated the feasibility of Raman microscopy for the molecular analysis with spatial information, and the Raman microscopy may provide an effective means to investigate the pathogenesis of NAFLD based on the characteristics of accumulated lipids.

Our findings in this study are that the lipids in LDs have the potential for complex distributions in terms of cholesterol and unsaturation degree of lipids. It is known that the activation of inflammatory cells is possibly induced by intake of LDs containing cholesterol accumulated in hepatocytes^19^. The unsaturation degree of lipids is also related to inflammation according to the difference in metabolic activity of lipids^29,30^. As a result, the complicated distributions of lipids in LDs may indicate the possible places where inflammation is likely to occur according to the distribution of cholesterol and unsaturation degree of lipids, possibly leading to the investigation of the pathogenesis of NALFD in terms of lipids. Furthermore, the potential of leading to liver-related complications can only be diagnosed after inflammation and fibrosis are well developed under the current pathological diagnosis. If the relationship between lipids and inflammation becomes clear, it will lead to the possibility of diagnosis at the earliest stage of NAFLD or before the onset of the inflammation and fibrosis. Thus, although further investigations are required, our findings will possibly provide the fundamental knowledge for elucidating the pathogenesis of NAFLD and establishing diagnostic methods in terms of lipids in the future.

Conventional bioimaging methods focusing on lipids are dye-based visualisation methods and imaging mass spectrometry; however, the molecular selectivity of the dye-based visualisation methods in lipid analysis is low because of the difficulty of the application of such as an immune-staining method or similar method of fluorescent proteins in the same manner of protein imaging. Therefore, the dye-based visualisation of lipids for NAFLD analysis is limited to morphological analysis. The imaging mass spectrometry is sensitive and selective in terms of lipid molecules and can clarify the details of lipid species, the length of acyl chains, and the degree of unsaturation. However, it is challenging to analyse microvesicular LDs or internal structures of LDs due to its low spatial resolution (> 10 µm). By contrast, Raman microscopy can achieve very high spatial resolution up to about half of the excitation wavelength, while obtaining molecular information via molecular vibrations. Although the Raman spectrum provides the partial molecular information such as the presence of CH_2_ structure, C=C double bond and steroid ring, Raman microscopy will be a powerful means to discuss the molecular species and structures of lipids such as cholesterol content via the C=C double bond and cholesterol content via steroid ring vibrations. Moreover, Raman microscopy has a potential for application in intraoperative use by using a surgical microscope or flexible optical fibre system^10,31^. Raman microscopy offers the advantages of fast, in situ, non-destructive and molecular vibration-based observation, enabling the diagnosis applications for NAFLD without any treatment such as fixation nor staining. Although further development of an intraoperative Raman microscopy system is required, our proposed approach may also provide a unique and powerful means for NAFLD diagnosis in the future.

## Conclusion

In conclusion, we demonstrated Raman spectral imaging of the liver tissues of NASH model mice. Our results revealed the feasibility of the Raman microscopy for the analysis of NAFLD based on the molecular features of accumulated LDs. Although further studies are required, our proposed approach may provide new insights into the investigation of the pathogenesis of NAFLD and the new development for NAFLD diagnosis based on accumulated lipids in the future.

## Materials and methods

### NASH model animal

In this study, we utilized a fatty liver of mice exhibited NASH for the evaluation of Raman microscopy in the investigation of NAFLD. The detailed induction protocol and histopathological characterisation of the NASH model mice used in this study were given in our previous study^32^. NASH model mice were obtained by feeding six-weeks-old male TSOD mice (Institute for Animal Reproduction, Ibaraki, Japan) on a high-fat/cholesterol/cholate diet (iHFC diet #5; Hayashi Kasei, Osaka, Japan) and purified water for 26 weeks and housed under normal conditions. The liver of the NASH model mice was excised after euthanasia. All procedures performed in studies involving animals were in accordance with the ethical standards of the institution at which the studies were conducted, and ethical approval was obtained from the Institutional Animal Care and Use Committee of Tokushima University, Japan (Approval number: T30-32).

### Sample preparation

The excised liver was immediately embedded in 4% sodium carboxymethyl cellulose compound and snap-frozen in liquid nitrogen. The samples were stored at − 80 °C until cryostat sectioning. The frozen samples were sliced into 5-µm thick sections with a cryostat microtome (Tissue Tek; Sakura, Tokyo, Japan). Since the compound-embedded liver was snap-frozen and kept frozen until the sectioning, the tissue embedding compound presumed not to penetrate to the evaluated area of the liver. Two serial sections were obtained for Raman and histopathological analysis. The sections for the Raman analysis were mounted on a slide glass without any fixation nor staining. The sections for histopathological analysis were fixed with 95% ethanol and were subjected to hematoxylin and eosin (HE) staining. The histopathological characterisation, such as the presence of LDs, hepatocytes and other histological features, was performed by a pathologist (K.T.), who is the co-author of this article. We obtained 12 sections from the livers of a total of two representative NASH model mice.

### Raman microscopy

Raman spectra and Raman spectral images were acquired with a home-built laser-scanning confocal Raman microscope with an imaging software (MwMapper, version 1.4.5; SicenceEdge Inc., Shizuoka, Japan, https://scienceedge.com). A single-mode frequency-doubled Nd:YAG laser (MSL-FN-532-S-100mW; CNI Laser, Changchun, China) operating at the wavelength of 532 nm was used as an excitation laser light. The excitation laser light was focused on a sample through a 10 × objective lens (CFI Plan Apo Lambda 10X, 10x, NA = 0.45; Nikon, Tokyo, Japan) or a 60 × objective lens (CFI Plan Apo Lambda 60XC, 60x, NA = 1.2; Nikon, Tokyo, Japan). The back-scattered Raman signal was collected with the same objective lens and detected by a spectrometer (IsoPlane 320, Princeton Instruments, Trenton, NJ, USA) with a cooled CCD image sensor (Pixis 400BR, − 70 °C, 1,340 × 400 pixels; Princeton Instruments, Trenton, NJ, USA). Raman spectrum from -30 to 3588 cm^−1^ was simultaneously obtained with a single exposure. Two-dimensional Raman spectral images were obtained by scanning the laser focus. The excitation laser power and the exposure time were 10 mW on the sample plane and 0.1 s, respectively.

### Spectral preprocessing

Raman shifts of all Raman spectra were calibrated by using the known bands of a calibration lamp (IntelliCal; Princeton Instruments, Trenton, NJ, USA). To extract the Raman spectrum from a broad fluorescence background, we applied a modified polynomial curve fitting method^33^. We estimated the autofluorescence component superposed on the Raman spectrum by calculating a modified least-squares 10-order polynomial curve with 100 iterations and then subtracted this polynomial from the raw spectra, as shown in Supplementary Fig. 1. The subtraction parameters were carefully adjusted to avoid the introduction of artefacts on the subtracted spectra. We confirmed that the artefacts in the subtracted Raman spectra were small enough for the analysis by comparing with the original spectra, as shown in Supplementary Fig. 1. Furthermore, we carefully confirmed that the Raman bands we focused on were originated from the liver tissues by comparing with that of a silica slide glass (Supplementary Fig. 2).

## Supplementary information

Supplementary Information.

## Data Availability

The data that support the findings of this study are available from the corresponding author upon reasonable request.
