# Supplementary material for: Molecular imaging analysis of microvesicular and macrovesicular lipid droplets in non-alcoholic fatty liver disease by Raman microscopy
Source: Sci Rep. 2020 Oct 29;10:18548. doi: 10.1038/s41598-020-75604-6 (PMC7596489; doi:10.1038/s41598-020-75604-6)
Supplement: Supplementary file 1 — Supplementary Information. [file 41598_2020_75604_MOESM1_ESM.pdf]

Molecular imaging analysis of microvesicular and macrovesicular lipid droplets in non-alcoholic fatty liver disease by Raman microscopy

Takeo Minamikawa[1,2,3,4*], Mayuko Ichimura-Shimizu[4,5], Hiroki Takanari[4,6], Yuki Morimoto[4,5], Ryosuke Shiomi[2], Hiroki Tanioka[2], Eiji Hase[1,4], Takeshi Yasui[1,2,4], and Koichi Tsuneyama[4,5,6]

[1]Department of Post-LED Photonics Research, Institute of Post-LED Photonics, Tokushima University, 2-1 Minami-Josanjima, Tokushima 770-8506, Japan.

[2]Graduate School of Technology, Industrial and Social Sciences, Tokushima University, 2-1 Minami-Josanjima, Tokushima, Tokushima 770-8506, Japan

[3]PRESTO, Japan Science and Technology Agency (JST), 2-1 Minami-Josanjima, Tokushima, Tokushima 770-8506, Japan.

[4]Research Cluster on "Multi-scale Vibrational Microscopy for Comprehensive Diagnosis and Treatment of Cancer", Tokushima University, 2-1 Minami-Josanjima, Tokushima, Tokushima 770-8506, Japan.

[5]Department of Pathology and Laboratory Medicine, Graduate School of Medical Sciences, Tokushima University, 3-18-15 Kuramoto, Tokushima, Tokushima 770-8503, Japan.

[6]Department of Interdisciplinary Researches for Medicine and Photonics, Institute of Post-LED Photonics, Tokushima University, 3-18-15 Kuramoto, Tokushima, Tokushima 770-8503, Japan.

*Correspondence to: Takeo Minamikawa, minamikawa.takeo@tokushima-u.ac.jp

Supplementary Figures

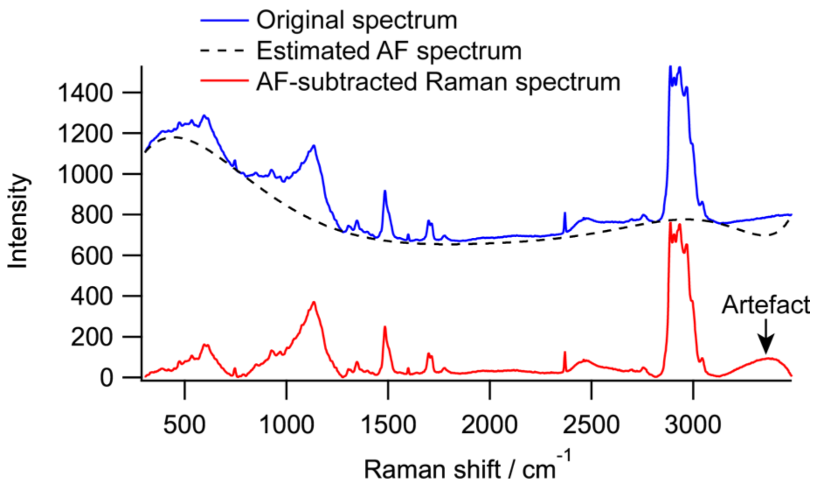

Supplementary Fig. 1 Modified polynomial curve fitting method to extract the Raman spectrum from a broad fluorescence background. Although an artefact was detected in the higher wavenumber region above 3100 cm^{-1}, the Raman spectrum from 600 to 1900 cm^{-1} and from 2700 to 3100 cm^{-1}, which is the Raman spectrum we focused on in this study, was extracted without artefacts from the original spectrum. AF, autofluorescence.

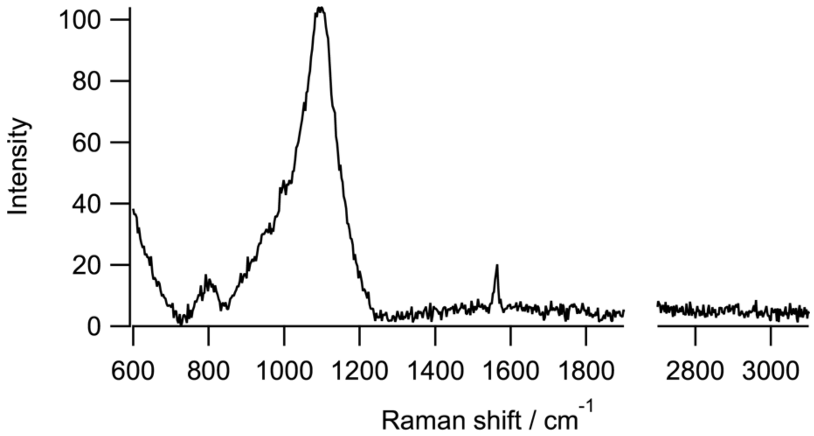

Supplementary Fig. 2 Typical Raman spectrum of a silica slide glass. The strong Raman bands of the silica slide glass were found below 1200 cm^{-1}. The Raman band at 1564 cm^{-1} represents the oxygen in the atmosphere. In the other region, no apparent Raman band was found.

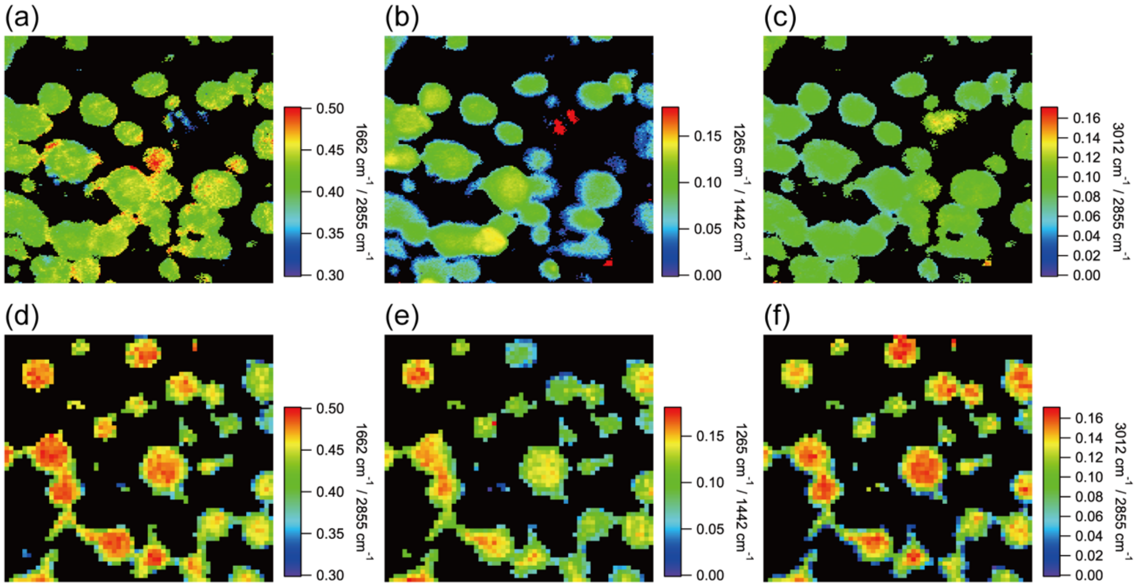

Supplementary Fig. 3 Intensity ratio imaging of various Raman bands indicating the unsaturation degree of LDs. Intensity ratio imaging of the macrovesicular LDs with the Raman bands of (a) 1662 against 2855 cm^{-1} as same as Fig. 3d, (b) 1265 against 1442 cm^{-1} and (c) 3012 against 2855 cm^{-1}. Intensity ratio imaging of the microvesicular LDs with the Raman bands of (d) 1662 against 2855 cm^{-1} as same as Fig. 4d, (e) 1265 against 1442 cm^{-1} and (f) 3012 against 2855 cm^{-1}.
